# Bilayer Poly(Lactic-co-glycolic acid)/Nano-Hydroxyapatite Membrane with Barrier Function and Osteogenesis Promotion for Guided Bone Regeneration

**DOI:** 10.3390/ma10030257

**Published:** 2017-03-03

**Authors:** Li Fu, Zhanfeng Wang, Shujun Dong, Yan Cai, Yuxin Ni, Tianshou Zhang, Lin Wang, Yanmin Zhou

**Affiliations:** 1 Department of Dental Implantology, School and Hospital of Stomatology, Jilin University, Changchun 130021, China; fuli1127@126.com (L.F.); supercarol324@163.com (Y.N.); thomas_1213@163.com (T.Z.); 2 Department of Neurosurgery, China-Japan Union Hospital of Jilin University, Changchun 130033, China; tattooreal@163.com; 3 VIP Integrated Department, School and Hospital of Stomatology, Jilin University, Changchun 130021, China; dsj@jlu.edu.cn (S.D.); wanglin1982@jlu.edu.cn (L.W.); 4 Department of Oral Mucositis, School and Hospital of Stomatology, Jilin University, Changchun 130021, China; caiyan2013@163.com

**Keywords:** GBR membrane, poly(lactic-co-glycolic acid), nano-hydroxyapatite, barrier function, osteogenesis, osteoblast

## Abstract

Guided bone regeneration (GBR) is one such treatment that reconstructs neo-bone tissue by using a barrier membrane to prevent the invasion of soft tissue and to create a space for guiding new bone growth into the bone defect. Herein, we report a novel functionally graded bilayer membrane (FGBM) for GBR application. To fabricate the novel membrane, the composites of poly(lactic-co-glycolic acid) and nano-hydroxyapatite were prepared by phase inversion for the dense layer and by electrospinning for another porous layer, and their corresponding properties were evaluated including surface morphology, mechanics, degradability, cell barrier function, and in vitro osteogenic bioactivity. The results showed that PLGA with 5% nHA in dense layer could meet the requirement of mechanical strength and have excellent barrier function even on condition of post-degradation. Furthermore, PLGA with 30% nHA in porous layer could achieve the good physical and chemical properties. In addition, 30% nHA incorporation would enhance the in vitro mineralization, and have superior capabilities of cell adhesion, proliferation and differentiation compared to other groups. Therefore, the designed FGBM could potentially serve as a barrier for preferential tissue ingrowth and achieve a desirable therapeutic result for bone tissue regeneration.

## 1. Introduction

Bone insufficiency or defects arising from tumor, trauma, or periodontitis frequently precludes the successful outcome of prosthodontics and dental implants [1]. During the past few decades several therapeutic approaches, including distraction osteogenesis [2], osteoinduction [3], osteoconduction [4,5] and guided bone regeneration (GBR) [6,7], have been utilized to increase bone volume. GBR technology is commonly used in dentistry and bone field. The basic principle of GBR involves the placement of mechanical barriers to protect blood clots and to isolate the bone defect from the surrounding connective tissue, thus providing bone-forming cells with access to a secluded space intended for bone regeneration [8,9,10,11]. According to this principle, GBR membranes would play a vital role in bone repair.

GBR membranes are grouped into two categories: non-resorbable membrane and resorbable membrane, according to their degradation characteristics. Non-resorbable membrane, such as titanium membrane and polytetrafluoroethylene (PTFE) membrane, has preferable clinical effect but with the need of secondary surgery for device removal [12,13]. Furthermore, the stiffness of non-resorbable membranes cause a higher number of exposures compared with resorbable membranes, which increase the risk of infection [14,15]. Therefore, non-resorbable membrane is now gradually replaced by resorbable membrane which includes natural polymers such as collagen and synthetic polymers such as poly(lactic-co-glycolic acid) (PLGA) [12,13]. The disadvantages of resorbable materials, however, are their low mechanical strength and unpredictable degree of resorption, which can significantly alter the amount of bone formation [9,16,17].

An ideal GBR membrane should address the requirements for biocompatibility, barrier action, space-making feature and clinical manageability [8]. A proper membrane surface structure can facilitate the proliferation and migration of bone cells, and accelerate bone formation in defect area [9]. In recent years, to meet the above requirements and promote osteogenesis, researchers have proposed the concept of functionally graded membrane, in which an asymmetrical membrane was prepared with different compositions and structures, toward bone tissue and epithelial tissue respectively [6,11,12,18].

PLGA attracts considerable attention because their degradation rate can be adjusted by altering the ratio of lactic to glycolic acids [9,17]. However, the disadvantage includes lack of osteoconductivity and local inflammatory response caused by their acidic degradation product [19]. To solve these problems, some researchers prepared calcium/phosphorus particles-loaded polymer membrane with excellent biocompatibility and osteoconductivity, which can enhance cell activity and neutralize acidic degradation products of polymer by ionic interaction [20,21]. Similar to natural bone mineral, hydroxyapatite (HA) is relatively easier to be identified by cells or biomacromolecules, which can improve the bioactivity and osteoconductivity of scaffolds [22]. Moreover, the release of calcium and phosphorus ions during the degradation of HA may be involved in bone metabolism to promote the formation of new bone [22,23]. Polymers combined with HA are expected to have better mechanics strength than pure synthetic polymers and improve structural integrity and flexibility over brittle glasses and ceramics for potential applications in bone repair and regeneration [23,24].

There are several methods of barrier membrane fabrication, including solvent casting [6,25], phase inversion [18,26], electrospinning [13,27], and so on. Membrane made by casting has the disadvantage of being unfavorable for cell adhesion due to its compact structure. Moreover, inhomogeneous particle distribution and poor mechanical strength are likely to occur when inorganic particles are added into the membrane [26]. Membrane, made by phase inversion method through solvent vaporization and subsequent immersion precipitation, can present an asymmetric structure including a loose layer and a dense layer [18]. However, non-uniform pore structure and poor dispersion of filler in matrix was still a problem. Electrospinning technique was generally used to fabricate micro- or nano-scaled fibers with large specific surface area and interconnected porous structure [28], which can facilitate osteogenesis and the function of osteoblast [13,27]. Recently, numerous studies have explored its use to generate fibrous scaffolds for tissue regeneration [12,13,23,24,29]. Nevertheless, pure electrospinning membrane lacks enough mechanical strength to serve as GBR membrane [30]. Furthermore, the fibrous matrix with high porosity level could not prevent the fibroblast infiltration.

Therefore, the objectives of this study were to: (1) develop a novel PLGA/nHA functionally graded bilayer membrane with different structures of the two surfaces by the combination of phase inversion and electrospinning for the first time; (2) investigate the surface morphology, mechanics, degradability, physical barrier function against cells, and in vitro osteogenic bioactivity; and (3) determine the optimum nHA/(PLGA + nHA) mass fractions of each layer. The following hypotheses were tested: (1) nHA incorporation into PLGA would not compromise the mechanical properties, matching commercial bilayer collagen membrane; (2) PLGA/nHA bilayer membrane could successfully impede the fibroblast infiltration; and (3) the more porous layer with higher contents of nHA would benefit and enhance bone tissue regeneration in vitro.

## 2. Experimental Section

### 2.1. Fabrication and Characterization of Phase Inversion Membrane

The dense layer was fabricated via a phase inversion method which described previously [26]. Poly(lactide-co-gly-colide) (PLGA, LA:GA = 75:25) with molecular weight of 100,000 was a gift donated by Changchun institute of applied chemistry (Changchun, China). In brief, PLGA was dissolved in N,N-dimethylformamide (DMF, Aladdin Chemical Co. Ltd., Shanghai, China) at a final PLGA mass fraction of 5%. Different contents of nHA (50–100 nm in length and 20–30 nm in width, Aladdin Chemical Co. Ltd.) was added in the mixture at nHA/(PLGA + nHA) mass fractions of 0%, 5%, 10% and 20%, and stirred for 2 h to ensure good nanoparticle dispersion. Afterwards, the solution was casted onto glass plate and spread slowly to form an even liquid film at an approximate thickness of 0.1 mm at room temperature. The glass plate was immersed in the water bath at 25 °C until the membrane entirely comes off. Subsequently, the obtained membrane was washed repeatedly with deionized water and dried in vacuum at 40 °C. This yielded a single layer phase inversion membrane (denoted “PIM”) with a smooth side (potentially towards the soft tissue) and relatively rough side (potentially serve as a base for the second layer), including four different types:

(1) PLGA phase inversion membrane without nHA (denoted PLGA PIM control);
(2) PLGA phase inversion membrane + 5% nHA (denoted PLGA PIM + 5 nHA);
(3) PLGA phase inversion membrane + 10% nHA (denoted PLGA PIM + 10 nHA);
(4) PLGA phase inversion membrane + 20% nHA (denoted PLGA PIM + 20 nHA).

The tensile properties of the four types of PIM were evaluated. The membranes were cut into rectangular samples (20 mm × 15 mm) and tested in dry state by an ultimate tensile test machine (3367, Instron, Norwood, MA, USA) with a crosshead speed of 10 mm/min at 25 °C (*n* = 6) [18]. The tensile strengths were calculated and recorded using a Blue Hill Systems.

The morphology of the PIM was investigated by an environmental scanning electron microscope (SEM; Model XL 30 ESEM FEG, Micro FEI Philips, Amsterdam, The Netherlands) equipped with energy dispersive X-ray (EDX) spectroscopy. A layer of gold was sprayed uniformly over the sample surfaces before the observation.

### 2.2. Barrier Function of Phase Inversion Membrane to Fibroblastic Cells

Since over 10% nHA incorporation into PLGA phase inversion membrane would compromise the mechanical properties, only PLGA PIM + 5 nHA was used for barrier function evaluation. The murine fibroblastic cell line L929 (American Type Culture Collection, Rockville, MD, USA) was applied for the evaluation. The in vitro barrier function of the phase inversion membrane to L929 cells was evaluated as described in a previous study [31]. PLGA PIM + 5 nHA was cut into a circle with a diameter of 25 mm to fix on a Cell Crown (Sigma-Aldrich, St. Louis, MO, USA) with 2-h UV light irradiation, and put into a 24-well plate without touching the bottom of the well. L929 cells were suspended in Dulbecco’s Modified Eagle Medium (DMEM) supplemented with 10% (*v*/*v*) fetal bovine serum (FBS; Gibco, Carlsbad, CA, USA) at a density of 4.0 × 10^3^ cells/mL. Afterwards, 1 mL of culture medium without cells was added to the well from the outside of the Cell Crown, and 1 mL of cell suspension was added onto the sample. In the process, it is important to keep the smooth surface of the membrane directly contacted with the cells. After incubated at 37 °C under a 5% CO_2_ atmosphere for 1 day and 3 days, the Cell Crown was removed and the bottom of the 24-well plate was stained by 4′-6-diamidino-2-phenylindole (DAPI, Sigma-Aldrich), and then observed under an inverted phase contrast microscope (TE2000-S, Nikon, Melville, NY, USA) to determine whether the cells had passed through the membrane to the bottom of the well or the culture medium. The barrier function of the PLGA PIM + 5 nHA after 30 days degradation in phosphate buffered saline (PBS) was also evaluated as above to investigate duration of barrier function against cells. The smooth surface of the membrane was also stained and observed for comparison. Twenty-four membrane samples were fabricated, yielding six samples per time point (*n* = 6).

### 2.3. Fabrication of Functionally Graded Bilayer Membrane

The phase inversion membrane with 5% nHA was verified with excellent mechanical properties and favorable barrier effect against fibroblast, which was described in detail later in Result section. Therefore, the second layer was fabricated on the base of relative rough surface of PLGA PIM + 5 nHA via electrospinning method as described in previous studies [32]. In brief, 1.2 g PLGA was added in 4 mL 1,1,1,3,3,3-Hexafluoro-2-propanol (HFIP, Aladdin Chemical Co. Ltd.), and the solution stirred vigorously until all polymer completely dissolved. Meanwhile, series content of nHA was added in 2 mL HFIP and ultrasonically mixed for 30 min. The two solutions mentioned above were mixed together and stirred continuously for 24 h before electrospinning to make the PLGA at a final concentration at 20%. The blend solution was loaded in a 10 mL plastic syringe connected to a stainless needle (inner diameter: 0.4 mm). The obtained fibers were collected on the rough surface of phase inversion membrane. The electrospinning parameters were as follows: electric field strength: 20 kV; air gap distance: 15 cm; flow rate of solution: 1 mL/h and all the experiments were conducted at room temperature in air. The obtained bilayer membrane was placed under vacuum for about 24 h at 25 °C to remove the residual solvent, finally yielding functionally graded bilayer membrane (FGBM).

### 2.4. The Surface Morphology of FGBM

Five different types of FGBM were prepared depending on the different nHA/(PLGA + nHA) mass fractions of 0%, 10%, 20%, 30% and 40%:

(1) PLGA functionally graded bilayer membrane without nHA (denoted FGBM control);
(2) PLGA functionally graded bilayer membrane + 10% nHA (denoted FGBM + 10 nHA);
(3) PLGA functionally graded bilayer membrane + 20% nHA (denoted FGBM + 20 nHA);
(4) PLGA functionally graded bilayer membrane + 30% nHA (denoted FGBM + 30 nHA);
(5) PLGA functionally graded bilayer membrane + 40% nHA (denoted FGBM + 40 nHA).

The tensile strength, surface and cross-section morphology of the five types of FGBM were studied by the protocols as described in Section 2.1. Six specimens per each kind of FGBM were tested for mechanical and SEM study (*n* = 6). Besides, a commercial collagen bilayer membrane Bio-Gide (Geistlich, Wolhusen, Switzerland) was also used as a commercial control in tensile strength evaluation.

### 2.5. In Vitro Biodegradation of FGBM

FGBM + 40 nHA had a relatively lower tensile strength and chaotic surface morphology, which was described in detail later in Result section. Therefore, FGBM + 40 nHA group was excluded in the following tests. Only four kinds of FGBM (Groups 1–4 in Section 2.4) were investigated in biodegradation measurement, simulated body fluid (SBF) immersion test and subsequent osteogenic bioactivity evaluation.

For in vitro degradation measurement, six specimens per each kind of FGBM were tested following previous studies (*n* = 6) [6,31]. Four kinds of FGBM were cut into rectangular strips of 8 mm × 5 mm and the starting weight (W_0_) was measured, and then all the samples were immersed in 10 mL PBS (0.1 M, pH = 7.4). The tubes were kept in a thermostable incubator maintained at 37 °C with a shaking speed at 100 cycles/min. The pH value in each group was measured once a week for up to 8 weeks. Meanwhile, the FGBM was carefully removed from the tube, rinsed with Milli-Q water for three times, dried until the weight underwent no further changes and then weighed (W_t_), the mass remaining ratio was calculated with the following formula:

Mass Remaining Ratio (%) = W_t_/W_0_ × 100%

### 2.6. SBF Immersion Test of FGBM

The formation of bone-like apatite on the surface of bilayer membranes was examined in SBF with ion concentrations nearly equal to those of human blood plasma [33]. The SBF was prepared by dissolving reagent grade NaCl, 0.350 g NaHCO_3_, 0.224 g KCl, K_2_HPO_4_·3H_2_O, MgCl_2_·6H_2_O, CaCl_2_, Na_2_SO_4_, and (CH_2_OH)_3_CNH_2_ into deionized water and titrated with 1 M HCl to the pH of 7.4 at 37 °C. Six samples per group were fabricated for SBF immersion test (*n* = 6). The bilayer membranes were trimed into square strips with the size of 1.5 cm × 1.5 cm and put in propylene tube with a cap containing 50 mL SBF, respectively. Afterwards, the tubes were kept in a thermostable incubator maintained at 37 °C and with a shaking speed at 100 cycles·min^−1^. The samples were taken out after 3 weeks, washed with deionized water and dried in vacuum. The morphology of samples incubated for 3 weeks were observed by SEM. Meanwhile, EDX analysis was also conducted to study calcium and phosphorus levels on bilayer membranes. Furthermore, the Ca^2+^ concentrations of SBF were also measured at 1, 3, 7, 14, and 21 days by atomic spectrophotometer (Shimadzu AA-360, Shimadzu, Kyoto, Japan).

### 2.7. Cell Adhesion on the FGBM

Murine osteoblast-like cells (MC3T3-E1 cells) were kindly supplied by the Medical Department of Jilin University, China. MC3T3-E1 cells have special behavior that is similar to primary calvarial osteoblasts. The medium for growth of the cell line was α modification of Eagle’s Minimum Essential Medium without ascorbic acid (α-MEM; Life Technologies, Carlsbad, CA, USA) containing 10% fetal bovine serum (FBS; Gibco, Carlsbad, CA, USA). The cells were placed under standard cell culture conditions, and the medium was changed every 2–3 days.

FGBM were irradiated by UV light for 2 h and placed into 24-well culture plates with the porous side facing up. Meanwhile, a sterile iron loop was used to fix the bilayer membrane. MC3T3-E1 cells were seeded on the porous surface at a density of 1.5 × 10^5^ cells/cm^2^ in a moist atmosphere of 5% CO_2_ at 37 °C. The cells cultured on the porous surface were counter stained by DAPI at 4 h, followed by observing under inverted phase contrast microscope (TE2000-S). The cell density D were measured and calculated as previously described. D_cell_ = N_cell_/A, where N_cell_ was the number of cell attached on the membrane surface, and A was the area of the image where N_cell_ was measured [34]. Six samples for each membrane type were fabricated for this measurement. Three randomly-chosen images for each sample were analyzed.

### 2.8. Cell Proliferation on the FGBM

The viabilities of MC3T3-E1 cells proliferated on the porous surface were assessed by using 3-(4,5-dimethyl-2-thiazolyl)-2,5-diphenyltetrazolium bromide (MTT) assay (Sigma-Aldrich) referred to the manufacturer’s protocol. MC3T3-E1 cells were seeded on the porous surface at a density of 1.5 × 10^5^ cells/cm^2^ according to a previous study [35]. At 1, 4 and 7 days of culture, the membranes were washed with PBS for three times to remove the cells that did not attach, respectively, and then 20 μL of MTT (5 mg/mL) was added and co-cultured for additional 4 h. The supernatant was removed and then the cell pellets were dissolved in 150 μL of dimethyl sulfoxide (DMSO, Sigma-Aldrich). The absorbance was determined using a Multi Skan plate reader (Lab systems, Helsinki, Finland) at a wavelength of 490 nm.

### 2.9. Cells Differentiation on the FGBM

The activity of alkaline phosphatase (ALP), a crucial component for initiating mineralization, was detected to evaluate the level of osteoblast early differentiation cultured on different kinds of FGBM. The FGBM samples were prepared and fixed in 24-well plates as described above in Section 2.7. MC3T3-E1 cells with a density of 1.5 × 10^5^ cells/cm^2^ were seeded on the porous side of the membranes in 24-well plates, and cultured for 1, 4 and 7 days. At each time point, the ALP activity was assessed by using *p*-nitrophenyl phosphate assay (pNPP, Sigma-Aldrich). In the presence of ALP, the pNPP could be conversed into *p*-nitrophenol (pNP) which was proportional to the ALP activity. Then the level of pNP production was determined from the absorbance at 405 nm using a microreader (Lab systems, Helsinki, Finland) [36].

### 2.10. Statistical Analysis

All data were checked for normal distribution with the Kolmogorov–Smirnov test. One-way analysis of variance (ANOVA) was performed to evaluate differences in tensile strength. Two-way ANOVA was used to assess differences in different kinds of FGBM and culture time. Post hoc multiple comparisons were performed using Tukey’s honestly significant difference test. Statistical analyses were performed by SPSS 19.0 software (SPSS, Chicago, IL, USA) at alpha of 0.05.

## 3. Results

**Figure 1:**
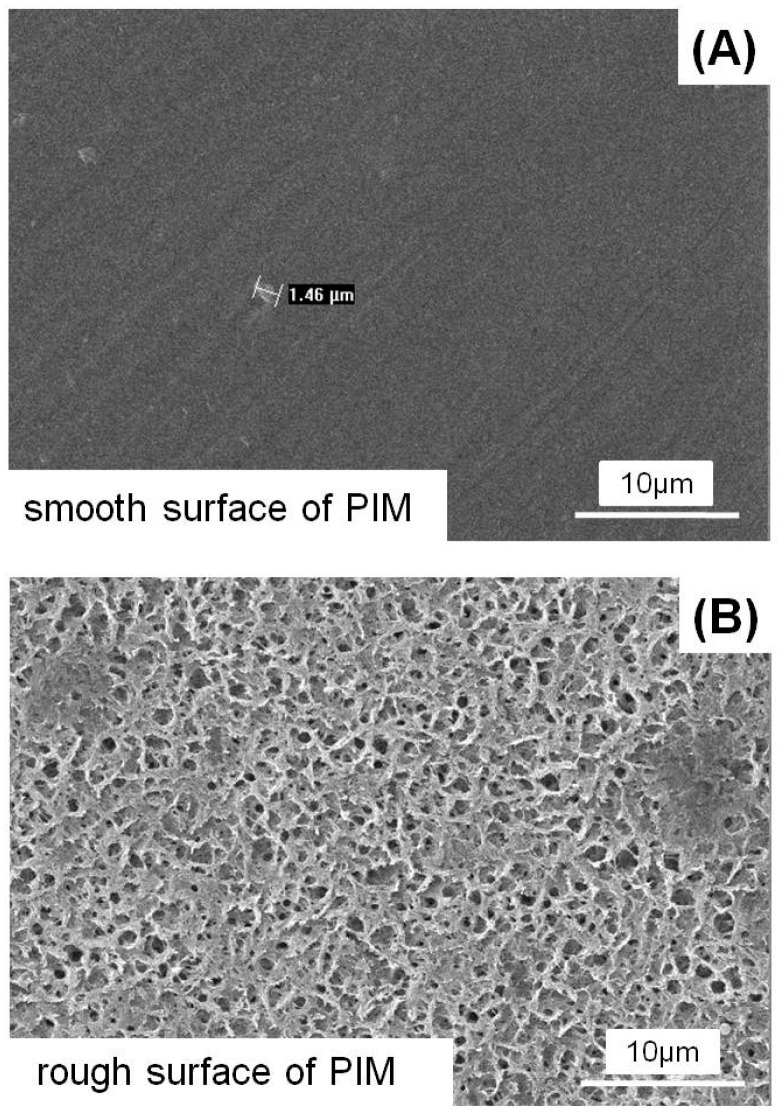
Representative SEM images of 5 wt % nHA loaded PLGA phase inversion membrane (PLGA PIM + 5 nHA), which presented an asymmetric structure: (**A**) Smooth surface; and (**B**) Rough surface with the average pore diameter being about 2–3 µm. SEM, scanning electron microscope. PIM, phase inversion membrane.

The tensile strengths of four types of PIM with different nHA contents are shown in Table 1. With the increase of nHA content, tensile strength of membrane first increased and then decreased. The 5% nHA incorporation would not compromise the tensile strength. However, over 10% nHA incorporation would significantly decrease the mechanical properties, compared to PLGA PIM + 5 nHA and PLGA PIM control (*p* < 0.05). In addition, the morphology of PLGA PIM + 5 nHA is plotted in Figure 1: the phase inversion membrane presented an asymmetric structure with one side being dense and smooth, and another porous with the average pore diameter of 2–3 µm.

**Figure 2:**
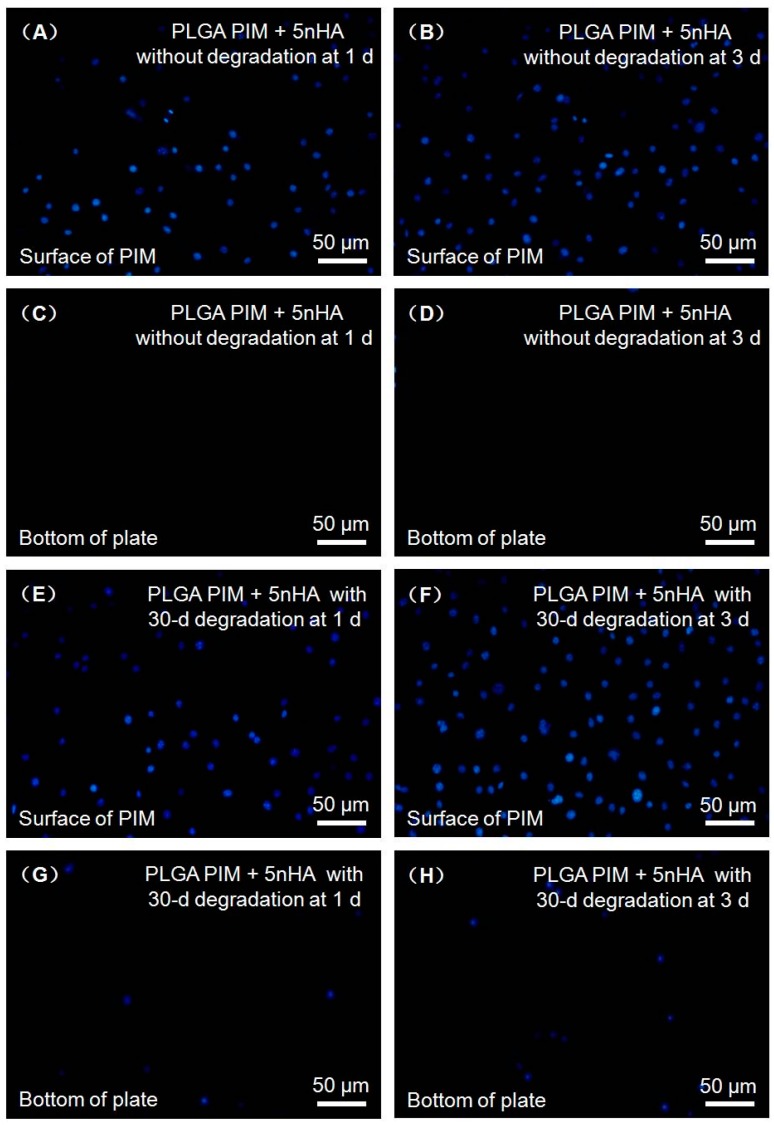
Representative fluorescent images of L929 cells cultured on the surface of PLGA PIM + 5 nHA: without (**A**–**D**) and with (**E**–**H**) degradation for one day (**A**,**C**,**E**,**G**) and three days (**B**,**D**,**F**,**H**). No cells were observed on the bottom of plate at one day and three days for PLGA PIM + 5 nHA without degradation. Few cells were observed on the bottom of plate at one day and three days for PLGA PIM + 5 nHA with 30-day in vitro degradation, which demonstrating PLGA PIM + 5 nHA exhibited an excellent cell barrier function. PIM, phase inversion membrane.

The physical barrier function against cells of the PLGA PIM + 5 nHA was tested in vitro by simulating penetration of fibroblast cells into the membranes. Figure 2 plots L929 cells on the surfaces of the membranes (Figure 2A,B,E,F) and bottom of the plate (Figure 2C,D,G,H) at one day (Figure 2A,C,E,G) and three days (Figure 2B,D,F,H) before (Figure 2A–D) and after (Figure 2E–H) degradation. Figure 2 shows that no cells penetrate to the opposite side of the membranes. Even undergoing degradation for 30 days, the membrane also could prevent L929 cells from penetration. Very few cells could reach the bottom of the well after three-days culturing.

Table 2 shows the tensile strength of FGBM of different nHA content and commercial control Bio-Gide. The FGBM resulted in similar tensile strength with that of PLGA PIM + 5 nHA alone (Data shown in Table 1). Nevertheless, there was a decline in the tensile strength of FGBM with the increase of nHA content in fiber layer. The results showed that more than 30% nHA incorporation into fiber layer would significantly decrease the tensile strength of FGBM (*p* < 0.05). However, FGBM + 30 nHA and FGBM + 40 nHA had the similar tensile strengths with the commercial control Bio-Gide (*p* > 0.05).

**Figure 3:**
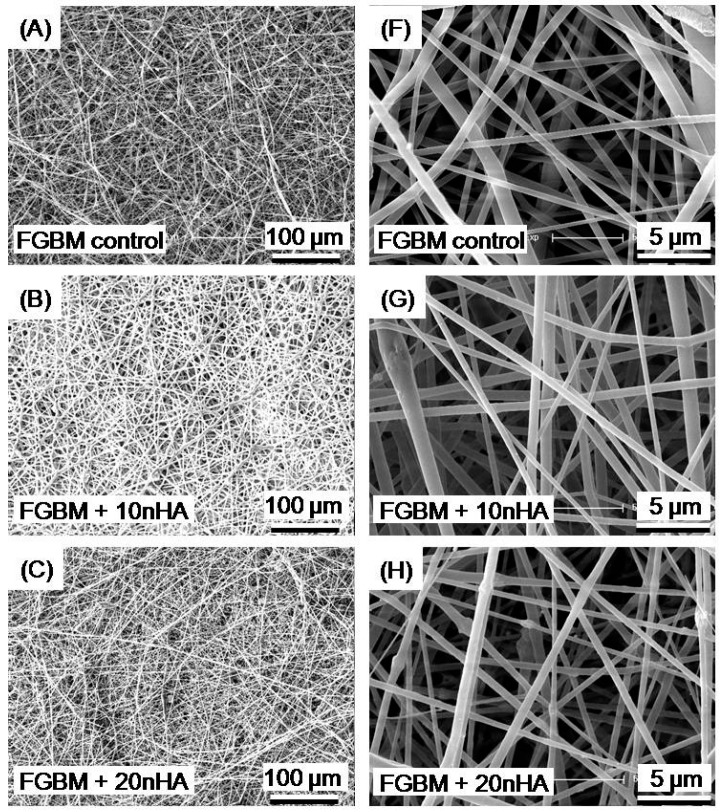

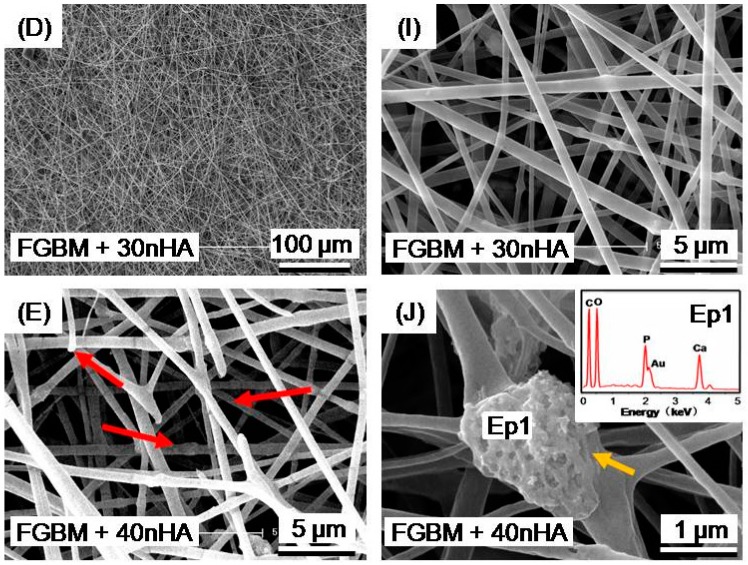
SEM micrographs of electrospun nanofiber membranes with different contents of nHA: (**A**,**F**) FGBM control (0% nHA); (**B**,**G**) FGBM + 10 nHA (10% nHA); (**C**,**H**) FGBM + 20 nHA (20% nHA); (**D**,**I**) FGBM + 30 nHA (30% nHA); and (**E**,**J**) FGBM + 40 nHA (40% nHA). All images presented a porous and interconnected structure composed of nano-sized fibers with a diameter distribution between 0.8 and 1.2 μm except FGBM + 40 nHA (**E**,**J**). The 40% nHA incorporation would lead to the frequent eletrospun fiber fracture and obvious nHA crystal agglomeration. The red arrows in (**E**) show the discontinuity of nanofibers. The yellow arrows in (**J**) show the presence of nHA particles agglomeration on the fiber surface. Ep1 is an EDX (energy dispersive X-ray) spectrum of selected point on crystal indicating presence of phosphorous (P) and calcium (Ca). SEM, scanning electron microscope. FGBM, functionally graded bilayer membrane.

As depicted in Figure 3, the nanofibers layers in FGBM control, FGBM + 10 nHA, FGBM + 20 nHA and FGBM + 30 nHA presented a porous and interconnected structure composed of nano-sized fibers without microbeads and with smooth surface on which no apatite crystals were detected, indicating that the nHA particles have been efficiently entrapped in the fibers with a diameter distribution between 0.8 and 1.2 μm. Nevertheless, the micro structure of eletrospun fiber layer of FGBM + 40 nHA, as shown in Figure 3E,J, indicated that 40% nHA addition would lead to the frequent eletrospun fiber fracture and obvious nHA crystal agglomerations, which were verified by the EDX spot analysis in Ep1. The EDX spectrum showed high Ca and P peaks, and the Ca/P ratio of the crystal was 1.71 (that was slightly high, considering it for the system error) approaching the stoichiometric value of HA of 1.67, which proved to be HA. The chaotic structures of FGBM + 40 nHA would decrease the mechanical properties and porosity of this composite, probably leading to lower osteogenic capabilities. Therefore, FGBM + 40 nHA was excluded from the subsequent experiments.

**Figure 4:**
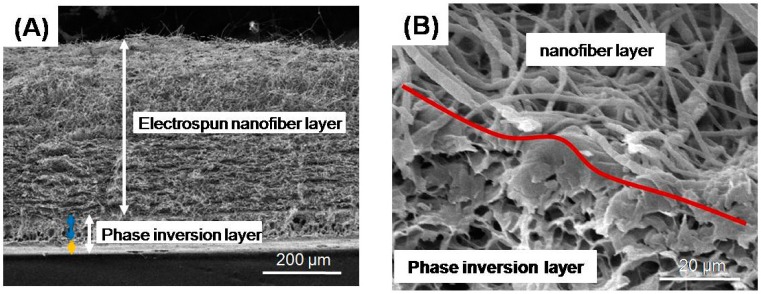
SEM images of cross section of FGBM + 30 nHA: (**A**) general view of the FGBM at a low magnification; and (**B**) electrospun nanofiber layer/phase inversion layer interface at a high magnification. The general thickness of FGBM was 500 µm with phase inversion layer being 100 µm (consisting of dense and porous layers marked by yellow and blue arrows, respectively) and nanofibers layer 400 µm. The red curve in (B) indicate the interface between phase inversion layer and electrospun fiber layer. The two layers were integrated closely without distinct boundary. SEM, scanning electron microscope. FGBM, functionally graded bilayer membrane.

Figure 4 plots the representative cross sectional SEM images of FGBM + 30 nHA. Images from three other kinds of FGBM were similar. The overall thickness of FGBM was about 500 µm with phase inversion layer being approximate 100 µm and nanofibers layer nearly 400 µm. The phase inversion layer also consisted of two structures: dense layer and porous layer, marked by yellow and blue arrows, respectively, in Figure 4A. The red curve in Figure 4B indicates the interface between phase inversion layer and electrospun fiber layer. The two layers were integrated so closely that no distinct boundary between them was found.

**Figure 5:**
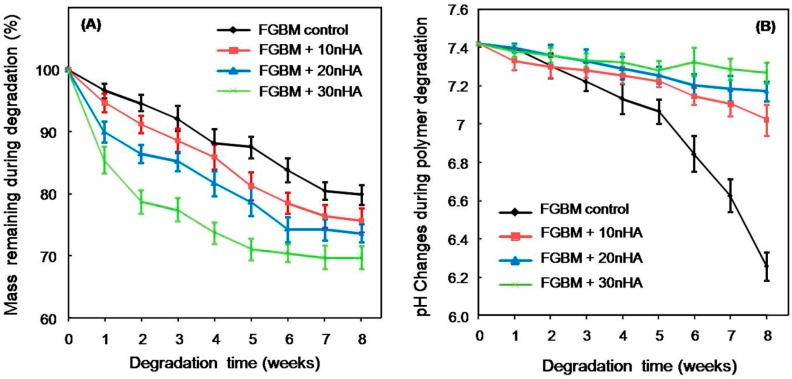
The mass remaining percentage (**A**); and solution pH changes (**B**) of the FGBM with different nanofiber layer during eight-week in vitro degradation (mean ± SD; *n* = 6). For all the time points, the encapsulation of nHA in the nanofibers increased the weight loss of FGBM. nHA incorporation would increase the stability of PBS solution. PBS, phosphate buffered saline. FGBM, functionally graded bilayer membrane.

Figure 5 plots the mass remaining percentage (Figure 5A) and pH variation (Figure 5B) of the FGBM in which nanofiber layer has different percentage of nHA. For all the time points, the encapsulation of nHA in the nanofibers increased the weight loss of FGBM. FGBM + 30 nHA showed a faster degraded speed than that of other groups. At the end of eight weeks, the remaining mass of FGBM + 30 nHA was approximate 70% of original mass. PLGA control exhibited a strong pH decreasing properties with degradation. nHA incorporation into FGBM would prevent the pH decline over eight weeks. More nHA incorporation into FGBM led to higher final pH values being detected at eight weeks. For example, the pH value only changed from 7.4 to 7.3 at the end of eight weeks for 30% nHA incorporation.

**Figure 6:**
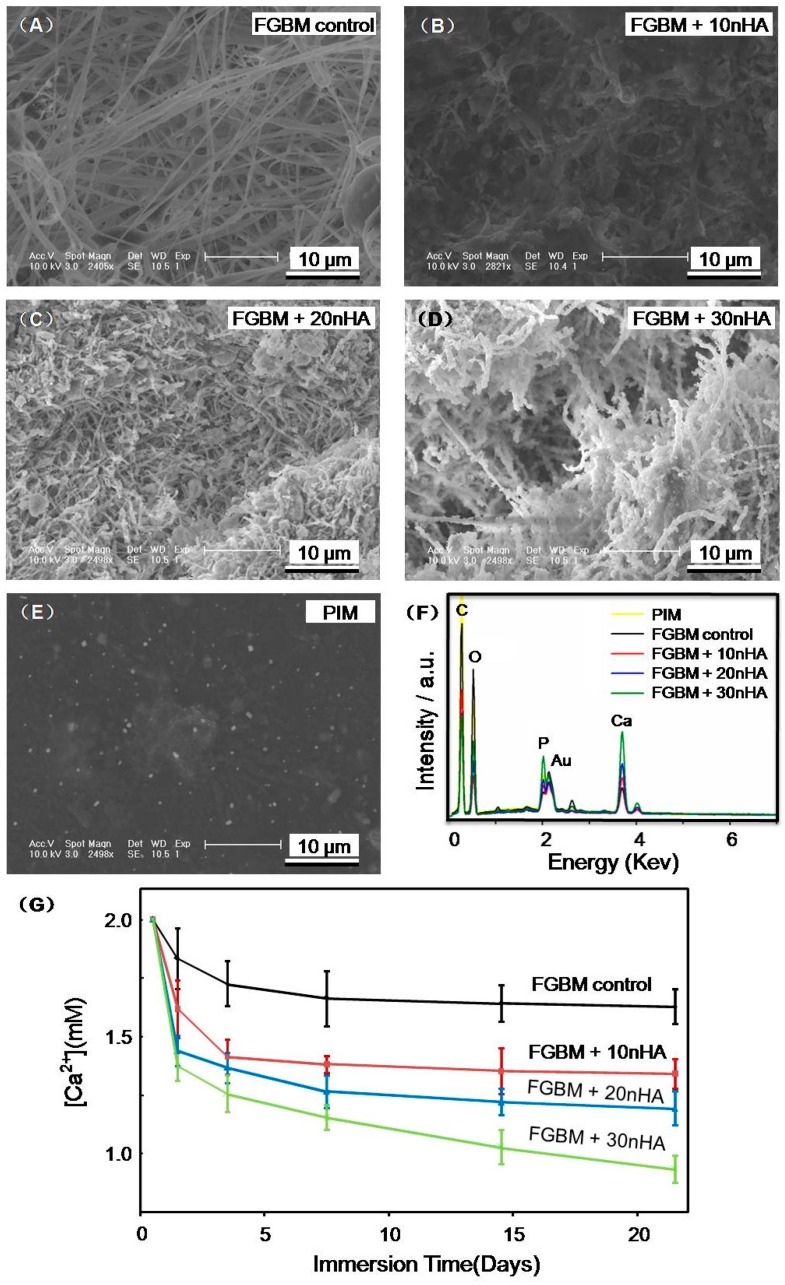
Apatite deposition on FGBM with different content of nHA after soaking in SBF for 21 days: (**A**) FGBM control; (**B**) FGBM + 10 nHA; (**C**) FGBM + 20 nHA; (**D**) FGBM + 30 nHA; and (**E**) PIM. The higher was the nHA content in the fibers, the more apatite was deposited. (**F**) EDX (energy dispersive X-ray) nalysis of the chemical elements of FGBM after immersion in SBF for 21 days. The calcium (Ca) and phosphorus (P) peaks were significant high on FGBM + 30 nHA, corresponding to the SEM images. (**G**) The quantitation of calcium ion concentration in the SBF during the immersion for 21 days (mean ± SD; *n* = 6). SBF, simulated body fluid. FGBM, functionally graded bilayer membrane. PIM, phase inversion membrane.

Figure 6 plots the SEM images of mineralization on the electrospun fiber layers of: (A) FGBM control; (B) FGBM + 10 nHA; (C) FGBM + 20 nHA; (D) FGBM + 30 nHA; and (E) PIM. The images clearly showed the deposition of apatite on the surface of eletrospun fiber layer with different content of nHA. Little apatite was observed on the PIM (smooth surface of the FGBM), as shown in Figure 6E. The higher contents of nHA were grafted in the eletrospun fibers, the more deposited apatite crystals were observed. Meanwhile, the chemical composition of FGBM was additionally investigated by EDX analysis after immersion in SBF. As seen in Figure 6F, EDX spectrum confirms the presence of calcium and phosphorus on the surface of FGBM. With the increase of nHA content in electronspun fiber layers, the carbon (C) and oxygen (O) peaks became weaker, while the calcium (Ca) and phosphorus (P) peaks were stronger, corresponding to the SEM images. Furthermore, the Ca^2+^ concentrations in the SBF with different groups also confirmed that FGBM + 30 nHA had the greatest amount of deposited apatite crystals during 21-day immersion, as shown in Figure 6G.

**Figure 7:**
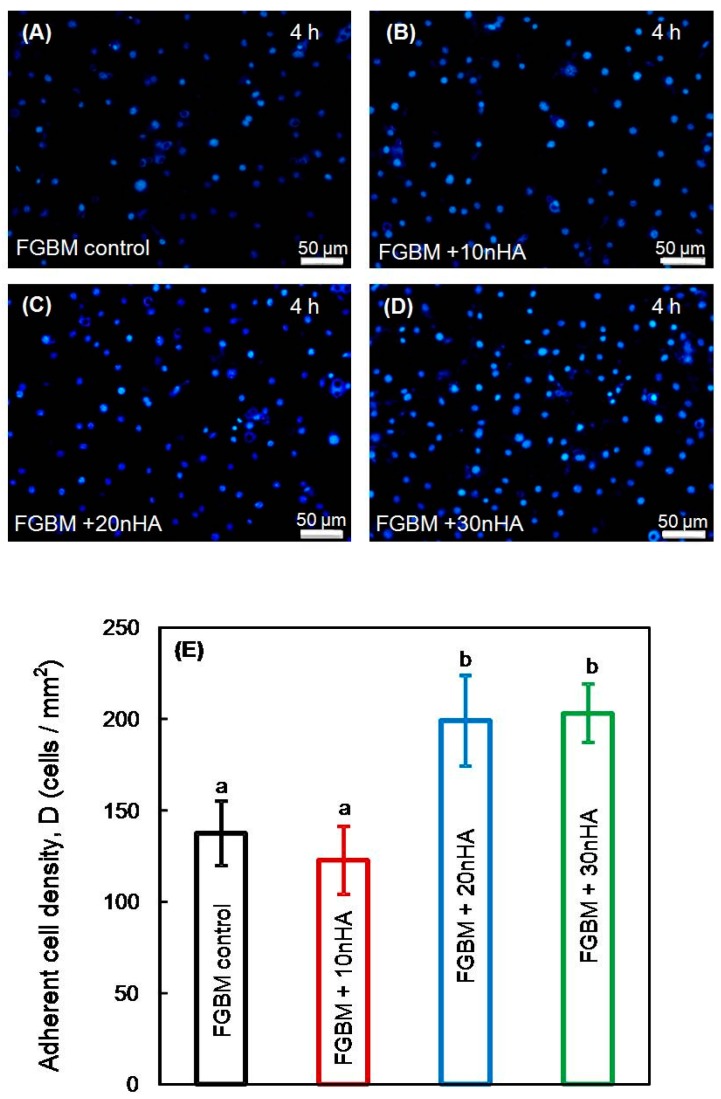
MC3T3-E1 cells adhesion onto the electrospun layer of different FGBM at 4 h: (**A**) FGBM control; (**B**) FGBM + 10 nHA; (**C**) FGBM + 20 nHA; and (**D**) FGBM + 30 nHA. (**E**) Cell density quantification of stained cells on the electrospun layer of different FGBM (mean ± SD; *n* = 6). Values with dissimilar letters are significantly different from each other (*p* < 0.05). FGBM, functionally graded bilayer membrane.

The adhesion of MC3T3-E1 cells onto the nanofiber surface of different kinds of FGBM was plotted in Figure 7A–D. The nucleuses of MC3T3-E1 cells were counter-stained with DAPI and the cell behavior on the different fiber layer was observed during the first 4 h of culture. The quantification in Figure 7E shows that FGBM + 20 nHA and FGBM + 30 nHA had significantly higher adherent cell density than that of FGBM control and FGBM + 10 nHA (*p* < 0.05), indicating nHA incorporation would enhance the osteoblast attachment onto the surface of the membrane.

**Figure 8:**
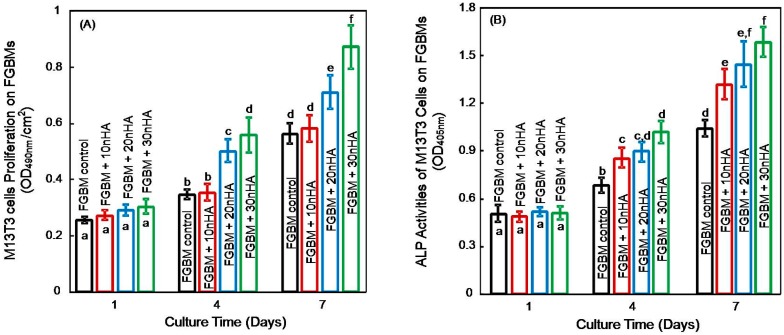
Cell proliferation and differentiation of MC3T3-E1 cells on different electrospun layer of FGBM at one, four and seven days: (**A**) MTT assay for proliferation assessmentl and (**B**) ALP activity of MC3T3-E1 cells for differentiation evaluation. In each plot, values with dissimilar letters are significantly different from each other (*p* < 0.05). MTT, 3-(4,5-dimethyl-2-thiazolyl)-2,5-diphenyltetrazolium bromide. ALP, alkaline phosphatase. FGBM, functionally graded bilayer membrane.

The proliferation and differentiation of MC3T3-E1 cells on the different kinds of FGBM are plotted in Figure 8. The cell viabilities at one day for all groups were similar. However, cells on the surface of FGBM + 20 nHA and FGBM + 30 nHA showed superior proliferation than that of FGBM control and FGBM + 10 nHA at four days. Furthermore, cells proliferation on FGBM + 30 nHA was significantly higher than that on FGBM + 20 nHA at seven days (*p* < 0.05). Early osteogenic differentiation of MC3T3-E1 cells on different types of FGBM was determined by ALP measurement (Figure 8B). At four and seven days, osteoblasts on FGBM with nHA incorporation appeared higher ALP activities than FGBM control without nHA. Cells on FGBM + 30 nHA had the highest ALP activity at seven days, which was 1.5-fold that on FGBM control without nHA (*p* < 0.05).

## 4. Discussion

The present study developed a novel PLGA/nHA functionally graded bilayer membrane with different structures and surfaces for the first time. The first dense layer was fabricated by phase inversion method. Base on this, a porous nanofiber layer was prepared via electrospinning method. For the dense layer, 5% nHA incorporation into PLGA would not compromise the mechanical properties and could perfectly prevent fibroblastic penetration, even in the process of membrane degradation. For porous layer, 30% nHA addition would not decrease the mechanical properties, exhibiting favorable and uniform porous structure. Moreover, the porous layer with PLGA and 30% nHA had excellent capabilities of cell adhesion, proliferation and differentiation in vitro. Therefore, this study showed that the designed bilayer membranes were promising for GBR therapy and other applications in bone tissue engineering.

GBR technology is commonly used for bone regeneration therapy, in which a membrane plays a vital role that can provide a secluded space around the bone defect for osteoblast migration and growth without the interference of fibroblast or epithelial cells [9,10]. Previous studies focused on the single layer membrane using in GBR therapy [37]. However, the membrane should not only perform the barrier function but also promote faster bone growth in clinical application. Therefore, a GBR membrane with two different surface morphologies, one towards soft tissue and another towards bone tissue, is highly desirable. Currently, asymmetric membrane with graded structure or composition to meet the local functional requirements has become one of the new trends [6,18,25,38]. The techniques for fabricating asymmetric membranes mainly include solvent casting [11], phase inversion [18,39] and electrospinning [12,27]. Leal et al. prepared a poly(d,l-lacticacid)/bioglass (PDLLA/BG) composite membrane with non-uniform distribution of BG by solvent casting method such that the BG rich side could stimulate bone ingrowth while the PDLLA rich side acted as a barrier [11]. However, the structure of this membrane was compact and unfavorable for cell adhesion. Ma et al. fabricated an asymmetric chitosan GBR membrane including a loose layer and a dense layer through phase inversion. However, in vivo results showed that a number of fibrous connective tissue appeared in some bone defects due to the poor marginal adhesiveness of the membranes [18]. Moreover, Bottino et al. developed a multi-layered membrane with different functional compositions by sequential electrospining. The membrane was composed of an interlayer serving as space maintenance and two functional layers in which bone-facing side was rich in nHA to facilitate osteogenesis and soft tissues-facing side metronidazole for anti-microbial [12]. Nevertheless, the barrier function to fibroblast of this pure electrospinning membrane was not tested.

The strategy of this study was to develop a PLGA/nHA bilayer membrane with graded structure and composition gradients simultaneously by a combination of phase inversion and electrospining, which was equipped with satisfactory barrier function, mechanical properties, biodegradation and osteoinduction for guiding bone regeneration. Phase inversion layer served as a barrier for soft tissue and a supporting for nanofibers, while electrospun nanofibers layer facing bone tissues facilitated the proliferation of osteoblast. Meanwhile, different contents of nHA were designed in each layer in order to improve mechanical strength and promote osteogenesis.

Medical devices made from the polymer, such as PLGA, have been associated with an inflammatory response, which may be caused by the accumulation of the acidic degradation product [25]. nHA is an excellent nanofiller candidate for resorbable polymers, which can neutralizes the acidic degradation products by ionic interaction between carboxylic acid groups of PLGA and calcium ions present in nHA [6,12,17,30]. Moreover, sufficient mechanical strength must be present to sustain mechanical loading without membrane collapse [12]. Previous studies indicated that a minimal addition of nHA to a polymer system increased mechanical strength compared with the neat blend system, while larger amounts of nHA decreased [24,40]. In the present study, the tensile strength of 5% nHA group was comparable with those recently reported in the literatures [18]. The PIM was a major contributor to the mechanical properties of FGBM, therefore, the PLGA PIM + 5 nHA was chosen for electrospining base.

The phase inversion membrane presented an asymmetric structure with one side dense and another porous. This structure was similar with that reported in a previous study [18]. The dense side of phase inversion layer prevents the ingrowth of fibrous connective tissue, and the porous side provides the supporting and binding sites for nanofiber layer. In the present study, the solvent starts to vaporize after the polymer solution casted on the glass plate. The homogeneous solution system was disturbed since the solvent on the surface vaporized faster than that of inside and consequently generated a poor polymer phase and a rich polymer phase. After the solvent gradually vaporized, the dense structure and the porous structure were formed, respectively [39]. In clinic practice, GBR membranes should maintain structure integrity until bone regeneration is achieved [41]. Previous research reported that during a GBR procedure, bone and/or periodontal ligament cell migration reached a peak at 2–7 days post-surgery. A decrease in mitotic activity to near normal levels was observed by the third week post-operative. This result implies that cells essential for regeneration usually arrive at the wound site in 3–4 weeks [42,43]. In the present study, the phase inversion membrane was verified to keep intact and exhibit satisfactory barrier function to L929 cell at least 30 days after degradation, therefore, allowing successful bone regeneration.

In order to further facilitate osteogenesis and activities of osteoblast, a porous layer produced by electrospinning was combined to phase inversion layer, resulting in a functionally graded bilayer membrane (FGBM). Electrospinning is a simple, versatile and well-documented method generally used to fabricate biomimetic nano-matrices with a high surface [12,13,44]. Our nanofiber layer of FGBM was made of PLGA/nHA composite with tremendous large surface area, similar with that in previous studies. Nevertheless, the nanofibers were not continuous and apt to be fractured when the nHA contents reached 40 wt %, which was mainly attributed to nHA particles agglomeration and its destruction of the continuity of polymer chains [29,44]. In addition, layer delamination as one of frequent interfacial failures was commonly detected due to the poor bonding between the different adjacent multiple layers [45]. In the present study, the interface section between phase inversion layer and electrospun layer was combined so tightly that it could bear tensile testing, which was owing to the highly compatible interface resulting from the rough binding sites of phase inversion layer and the same component in the two layers. Additionally, large amounts of nHA in nanofibers decreased the mechanical strength of FGBM, which is consistent with those previous reports [40]. This was probably due to the increased brittleness, and the disturbance to the continuous phase of polymer matrix [26,27]. However, FGBM + 40 nHA was still matched with that of Bio-Gide and satisfied the mechanical requirement for membranes as report earlier [12,18].

Regarding the clinical demand of GBR membrane, the biodegradation rate of membrane should match the healing or regeneration process [45]. In addition, the degradation products need to be nontoxic [46]. In this study, in vitro degradation results of the FGBM showed that the nHA was essential for degradation, which could accelerate the biodegradation rate of the membranes due to the increase in hydrophilicity and neutralize against acidic degradation products of PLGA [20,21]. Among the four membranes, FGBM + 30 nHA showed appropriate biodegradation rate and stable pH value which was suitable for new bone formation. Moreover, the nanofiber layer of FGBM + 30 nHA showed excellent capabilities of calcium collection and bone-like apatite formation, probably attributing to the rough surface morphology of electrospun layer and the incorporation of nHA particles acting as nucleation sites in SBF [30,32]. The new bone-like apatite on the materials surface could predict that the materials would exhibit a good bone bonding behavior, resulting in excellent osteoconductivity in vivo as GBR membranes [47,48].

The osteoblastic activities on the FGBM are important and determinant for the quality and suitability of the GBR membrane. Generally, in the cloned mouse calvarial cell line MC3T3-E1, a period of rapid cell division is followed by a transitional period that is characterized by deposition of type I collagen-rich extracellular matrix and an initial rise in alkaline phosphatase activity. Later, a third stage occurs, beginning approximately two weeks after plating, which is characterized by further increases in ALP activity, expression of osteocalcin, and mineral deposition [49]. In our study, nanofiber layers of FGBM could be regarded as an analogue of the extracellular matrix [50], exhibiting satisfactory cytocompatibility. In addition, the high contents of inorganic component had a positive impact in the adhesion, proliferation, differentiation of MC3T3-E1 cells on membranes, which was in accordance with previous studies [11,32,51,52]. In those studies, the incorporation of nHA increased the hydrophilicity [32,51] and adsorbed some adhesive proteins such as vitronectin and fibronectin from the serum, and thus enhanced the protein adsorption with the subsequent binding of the osteoblast precursor to the nHA [52]. Then, nHA might be exposed to the fiber surface during degradation, producing a rough nanofiber surface, resulting in the faster cell proliferation and the extracellular matrix production [53]. Furthermore, previous experimental studies have revealed that the expression of typical bone differentiation markers could be triggered by the incorporation of calcium phosphates into the cell culture environment and increasing the roughness of the cell culture substrate [27,36,54,55]. Regarding the cellular responses to the membranes, the osteoblastic activity was significantly higher on FGBM + 30 nHA than that on the other membranes, indicating that the membrane could recruit osteoblastic cells into the bone defect area, and the higher contents of nHA might also stimulate the cells to undergo an osteogenic process.

The present study successfully developed a designed functionally graded bilayer membrane via phase inversion and electrospinning methods, yielding excellent fibroblastic barrier function and favorable osteogenic effects. Due to the possible regenerative differences between in vitro and in vivo, further study should investigate the osteogenic effect of this novel construct as a GBR membrane in animal studies.

## 5. Conclusions

The present study synthesized a novel PLGA/nHA functionally graded bilayer membrane with different structures and surfaces, and achieved excellent fibroblastic barrier function and favorable osteogenesis effect for the first time. The results validated the hypotheses, demonstrating that up to 30% nHA incorporation into PLGA would not compromise the mechanical properties of the membrane which was comparable with the commercial control. Furthermore, PLGA/nHA bilayer membrane could successfully impede the fibroblast infiltration, even bearing 30-day degradation. Moreover, the more porous layer and higher contents of nHA would enhance osteoblast attachment, proliferation and differentiation in vitro, compared to control group. The PLGA/nHA functionally graded bilayer membrane may open a new direction for regenerative bone treatment and has potential to be applied in hard tissue regeneration.

## Figures and Tables

**Table 1:** Means (Standard deviations) of tensile strength of single layer phase inversion membrane (PIM) with different composition (*n* = 6) *.

Types of PIM	Tensile Strength (MPa)
PLGA PIM control	2.90 (0.31) ^a^
PLGA PIM + 5 nHA	3.55 (0.45) ^b^
PLGA PIM + 10 nHA	2.82 (0.32) ^a^
PLGA PIM + 20 nHA	2.03 (0.11) ^a^

* Differences among groups with different superscript letters are statistically significant (*p* < 0.05).

**Table 2:** Means (Standard deviations) of tensile strength of functionally graded bilayer membrane (FGBM) with different composition (*n* = 6) ^#^.

Types of FGBM	Tensile Strength (MPa)
FGBM control	3.80 (0.59) ^a^
FGBM + 10 nHA	3.59 (0.44) ^a^
FGBM + 20 nHA	3.35 (0.30) ^a,b^
FGBM + 30 nHA	3.18 (0.28) ^a,b^
FGBM + 40 nHA	3.09 (0.32) ^b^
Commercial control Bio-Gide	3.06 (0.24) ^b^

^#^ Difference among groups with different superscript letters is statistically significant (*p* < 0.05).
